# Estudio del metabolismo lipídico en niños aragoneses con sobrepeso/obesidad vs. niños normopeso

**DOI:** 10.1515/almed-2024-0113

**Published:** 2025-01-06

**Authors:** José Cuenca Alcocel, Lorena Villalba-Heredia, Inés Martínez Redondo, Alba Gallego Royo, José A. Casajús, José M. Arbonés-Mainar, Pilar Calmarza

**Affiliations:** Servicio de Bioquímica Clínica, Hospital Obispo Polanco, Teruel, España; GENUD Research Group, PhD Student, Universidad de Zaragoza, Zaragoza, España; 16488 Servicio de Pediatría, Hospital Universitario Miguel Servet, Zaragoza, España; 16488 Servicio de Medicina Preventiva, Hospital Universitario Miguel Servet, Zaragoza, España; GENUD (Growth, Exercise, Nutrition and Development) Research Group, Universidad de Zaragoza, Instituto de Investigación Sanitaria Aragón (IIS Aragón), Zaragoza, España; Centro de Investigación Biomédica en Red de Fisiopatología de la Obesidad y Nutrición (CIBEROBN), Instituto de Salud Carlos III, Madrid, España; Departamento de Fisiatría y Enfermería, Facultad de Ciencias de la Salud y el Deporte, Universidad de Zaragoza, Zaragoza, España; %2016488 Adipocyte and Fat Biology Laboratory (AdipoFat), Unidad de Investigación Transversal, Hospital Universitario Miguel Servet , Instituto de Investigación Sanitaria (IIS) Aragón, Zaragoza, España; Instituto Aragonés de Ciencias de la Salud (IACS), Zaragoza, España; CIBER Fisiopatología Obesidad y Nutrición (CIBERObn), Instituto Salud Carlos III, Madrid, España; %2016488 Servicio de Bioquímica Clínica, Hospital Universitario Miguel Servet , Isabel la Católica, Zaragoza, España; miembro de las Comisiones de Estrés Oxidativo y Lipoproteínas y Enfermedades vasculares de la SEQC^ML^, Centro de Investigación en Red en Enfermedades Cardiovasculares (CIBERCV), Universidad de Zaragoza, Instituto de Investigación Sanitaria Aragón (IIS Aragón), Zaragoza, España

**Keywords:** apolipoproteína A1, colesterol, lípidos, niños, obesidad, triglicéridos

## Abstract

**Objetivos:**

La obesidad y el sobrepeso en la infancia y/o adolescencia han aumentado considerablemente en los países europeos, durante los últimos años, representando actualmente un importante problema de salud pública mundial. El objetivo de este estudio es la detección precoz en un grupo de niños con sobrepeso/obesidad (8–12 años) de las alteraciones metabólicas que pueden conducirles, incluso en edades tempranas, a alteraciones en el metabolismo de la glucosa y/o enfermedad cardiovascular.

**Métodos:**

Se estudiaron una serie de parámetros del metabolismo lipídico y de control metabólico, en un grupo de 61 niños y niñas con sobrepeso/obesidad y en un grupo de 45 niños y niñas sanos, normopeso, de edades comprendidas, todos ellos, entre 8 y 12 años, comparando los resultados obtenidos.

**Resultados:**

Se encontraron concentraciones más elevadas en el grupo de niños con sobrepeso/obesidad, respecto al de niños normopeso, en: triglicéridos e insulina; y más bajas en: colesterol HDL y apolipoproteína A1. El cociente apolipoproteína B/apolipoproteína A1, el índice triglicéridos-glucosa y el índice HOMA fueron más elevados y la ratio colesterol LDL/apolipoproteína B más baja en los niños con sobrepeso/obesidad.

**Conclusiones:**

Según nuestros resultados, la obesidad a edades tempranas (8–12 años) afecta ya a la concentración de los parámetros lipídicos, habiéndose encontrado un perfil lipídico más aterogénico con mayor concentración de partículas remanentes y partículas LDL pequeñas y densas, mayor insulinoresistencia y mayor riesgo de desarrollar diabetes mellitus de tipo 2  y/o enfermedad cardiovascular en los niños con sobrepeso/obesidad, al compararlos con los normopeso.

## Introducción

La obesidad y el sobrepeso en la infancia y/o adolescencia han aumentado considerablemente en los países europeos durante los últimos años [1], representando actualmente un importante problema de salud pública mundial.

Según los criterios de la International *Obesity Task Force* (IOTF) [2], se considera obesidad infantil cuando el índice de masa corporal (IMC) es superior a los puntos de corte definidos por Cole et al. para un equivalente de 30 kg/m^2^ y sobrepeso cuando es superior al punto de corte para el equivalente de 25 kg/m^2^.

La obesidad está influenciada por factores tanto genéticos como ambientales [3] y tanto la alimentación como la actividad física juegan un papel fundamental en el crecimiento y la regulación del peso corporal. Así, una mala alimentación y la falta de actividad física, entre otros factores, puede conducir a la aparición de obesidad infantil y al inicio de la formación de la placa arteriosclerótica. Además, todo esto puede provocar la aparición en la edad adulta, incluso en edades tempranas, de trastornos en el metabolismo de la glucosa como la diabetes mellitus tipo 2 (DM2), alteraciones lipídicas o enfermedad cardiovascular (ECV) [4]. El sobrepeso o la obesidad contribuyen al desarrollo del “síndrome metabólico” (SM) en adultos y en niños [5], con la aparición de problemas como la enfermedad del hígado graso no alcohólico, la hipertensión, enfermedades coronarias y problemas osteomusculares, considerándose además un factor de riesgo para el desarrollo de algunos tipos de cáncer, así como de problemas escolares y psicológicos.

Según el estudio ALADINO 2019 [6] y algunos otros estudios [7], 8] pese a que parece que en los últimos años la prevalencia en España de la obesidad y el sobrepeso en la edad infantil se ha estancado, esta se sitúa en torno al 20 % en edades comprendidas entre los 6–12 años. Además, hay que tener en cuenta que España es actualmente el segundo país de Europa con mayor prevalencia de sobrepeso y obesidad infantil [9] y considerando su distribución según sexo, el porcentaje de sobrepeso es mayor en las niñas y el de obesidad en los niños.

Diversos estudios poblacionales [10] han puesto de manifiesto que la prevalencia de la dislipemia en los niños y adolescentes con sobrepeso y obesidad oscila entre el 20 % y más del 40 %, respectivamente, siendo superior a la que presentan los niños y adolescentes delgados o con peso normal que se sitúa en torno al 8–20 % [11].

Sin embargo, los estudios sobre dislipemia realizados en niños con obesidad o sobrepeso en nuestra población y aquellos que evalúan los parámetros metabólicos y hormonales son escasos, habiéndose encontrado en algunos de ellos que la obesidad puede tener influencia en la concentración de algunas hormonas como la hormona del crecimiento, insulina [12], 13], leptina y parathormona intacta (PTHi).

El objetivo de este estudio ha sido evaluar en niños obesos o con sobrepeso, de 8–12 años, de la comunidad Autónoma de Aragón, una serie de parámetros lipídicos básicos, apolipoproteínas A1 y  B, lipoproteína (a) (lp(a)) y metabólicos (insulina, resistencia a la insulina), comparando los resultados obtenidos con los de un grupo de niños normopeso, de edades similares.

## Materiales y métodos

Se trata de un estudio comparativo, en el que comparamos un grupo de niños con sobrepeso u obesidad con un grupo de niños normopeso, dentro de un marco observacional, que fue realizado en Zaragoza, Aragón, España. El grupo de niños con sobrepeso u obesidad estaba formado por 61 niños y niñas con sobrepeso u obesidad, siguiendo los puntos de corte definidos por Cole et al. [2]. Dichos niños procedían del estudio Exergames, llevado a cabo en la Universidad de Zaragoza, registrado en clinicaltrials.gov (con número de identificación NCT04418713), y aprobado por el Comité de Ética de Investigación del Gobierno de Aragón (certificado nº 11/2018, CEICA, Zaragoza, España). El estudio de Videojuegos Activos o también conocido como “Exergames” [14] buscaba conocer los efectos de un programa de ejercicio de Videojuegos Activos y Ejercicio Multicomponente en niños y niñas prepúberes con sobrepeso u obesidad. Para el grupo de niños normopeso, se partía de 59 niños y niñas normopeso, previamente a ser sometidos a una cirugía de importancia menor (criptorquidia, fimosis, traumatológica, entre otros) en el cual, las analíticas se realizaron antes de la cirugía, y tras revisión de las historias clínicas, se excluyeron aquellos que presentaban alguna patología, así como los que presentaban sobrepeso u obesidad, empleando los criterios de clasificación de la IOTF [2].

Tras aplicar estos criterios el grupo de niños normopeso, quedó constituido por 45 niños.

**Figura 1:**
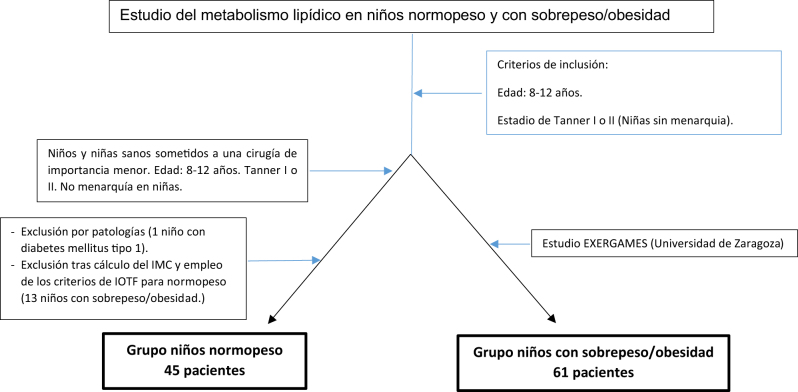
Selección de niños normopeso y niños con sobrepeso/obesidad.

En ambos grupos se seleccionaron niños/as de edades comprendidas entre los 8 y 12 años, procedentes de la Comunidad Autónoma de Aragón, que no hubieran iniciado el desarrollo puberal ni la menarquia en las niñas (estadios I y II de Tanner) y que no presentaran patologías (enfermedades metabólicas, crónicas, infección aguda, anorexia nerviosa) o tratamientos que pudieran influir en los parámetros del estudio. La selección de los grupos de estudio se esquematiza en la Figura 1.

En primer lugar, se informó a los padres del estudio a realizar, presentándoles toda la información por escrito, así como el documento del consentimiento informado, el cual fue firmado por todos ellos. A continuación, se realizó una breve encuesta sobre datos epidemiológicos y  clínicos, medición de las variables antropométricas: peso, talla e índice de masa corporal (IMC), una exploración física y una analítica en ayunas, donde se determinaron los parámetros de interés para la evaluación del metabolismo lipídico y el perfil metabólico.

Las muestras de sangre se extrajeron a primera hora de la mañana, tras un ayuno nocturno de 8 horas, en tubos con gel separador y se determinaron en suero los parámetros lipídicos básicos, colesterol total (CT), colesterol HDL (cHDL) y triglicéridos (TG) en un Autoanalizador AU 5800 (Beckman Coulter Miami, FL, EE.UU.), colesterol LDL (cLDL) mediante la fórmula de Friedewald, apolipoproteína A1 (Apo A1), apolipoproteína B (Apo B) y lp(a) en un Autoanalizador Immage (Beckman Coulter Miami, FL, EE.UU.) por inmunonefelometría e insulina en un equipo Cobas e411 (Roche Diagnostics, España).

Además, se calcularon los cocientes Apo A1/Apo B, TG/cHDL y cLDL/Apo B, índice triglicéridos-glucosa (TyG), mediante la fórmula: Ln (triglicéridos (mg/dL) × glucemia (mg/dL)/2); el número de partículas remanentes, mediante la fórmula: colesterol total – cHDL – cLDL, el índice de resistencia a la insulina (HOMA), mediante la fórmula: insulina (μU/mL) × glucemia (mmol/L)/22,5  y el índice cuantitativo de verificación de la sensibilidad a la insulina (QUICKI), mediante la fórmula: 1/(log insulina (µU/mL) + log glucosa (mg/dL)). Para el análisis estadístico se utilizó el programa IBM SPSS Statistics versión 26.0.

En primer lugar, tanto para los parámetros antropométricos como para los parámetros bioquímicos, para estudiar la distribución de las variables cuantitativas se utilizó el test de Kolmogorov-Smirnov con modificación de Lilliefors (KSL). En el caso de que siguieran una distribución normal (KSL, p>0,05) se emplearon para su descripción la media y la desviación estándar y en el caso de que las variables cuantitativas no siguieran una distribución normal (KSL, p≤0,05) la mediana y el rango intercuartílico.

Para la comparación entre los dos grupos de los parámetros antropométricos y bioquímicos se utilizó la prueba t-Student o el test de Welch, cuando se trataba de una distribución normal, dependiendo respectivamente de si las varianzas en ambos grupos eran homogéneas o no y el test U-Mann Whitney cuando se trataba de una distribución no normal.

Se realizó, también, un análisis estadístico similar para estudiar la idoneidad de los dos grupos de estudio en cuanto a edad, talla e IMC, comprobando si eran comparables en cuanto a estos parámetros.

Con el fin de estudiar la influencia del sexo en los grupos de niños con sobrepeso/obesidad y normopeso, se dividieron en dos subgrupos cada uno, según el sexo, quedando formado el subgrupo de niños con sobrepeso/obesidad por 34 niños y 27 niñas y el de normopeso por 29 niños y 16 niñas y, utilizando los test descritos anteriormente, se determinó si había diferencias significativas entre los distintos parámetros o índices estudiados en ambos subgrupos, según el sexo.

También se subdividió el grupo de niños con sobrepeso/obesidad en niños con sobrepeso y niños con obesidad, empleando los puntos de corte definidos por la IOTF, quedando un subgrupo formado por 40 niños/as obesos y otro por 21 niños/as con sobrepeso y empleando los test anteriores se determinó si había diferencias significativas entre los distintos parámetros o índices estudiados en ambos subgrupos.

Para estudiar la correlación de los parámetros bioquímicos con el IMC, la edad y el sexo (estos dos últimos distinguiendo entre niños normopeso y con sobrepeso/obesidad), primero se aplicó el test de KSL a la totalidad de la muestra para comprobar la normalidad de las variables, utilizando posteriormente el coeficiente de correlación de Pearson cuando las variables seguían una distribución normal, el de Spearman cuando no la seguían y el de Tau-b de Kendall cuando la variable era dicotómica (como en el caso del sexo). El nivel de significación estadística para todos los test estadísticos empleados se estableció a partir de un valor p≤0,05.

## Resultados

Los datos antropométricos del grupo de niños/as con sobrepeso/obesidad y los del grupo de niños/as normopeso se muestran en la Tabla 1. En cuanto a la edad no se encontraron diferencias estadísticamente significativas entre ambos grupos y en la proporción de niños y niñas los resultados también fueron comparables entre ambos grupos (55,7 % de niños en el grupo de sobrepeso u obesidad frente a 64,4 % de niños en el grupo normopeso, p=0,367).

**Tabla 1:** Parámetros antropométricos en niños normopeso y niños con sobrepeso/obesidad.

	Sobrepeso/Obesidad (n=61, 34 niños y 27 niñas)^d^			Normopeso (n=45, 29 niños y 16 niñas)^d^			Significación estadística (test)	Valor p test de Levene
	Total	Límites	Pruebas de normalidad^c^	Total	Límites	Pruebas de normalidad^c^		
Edad, años	10,1 ± 0,9^a^	(8,4–12,2)	0,200	10,1 ± 1,1^a^	(8,4–12,0)	0,200	0,912 (Welch)	0,014
Peso, kg	55,4 (40,6–, 70,2)^b^	(33,4–89,1)	0,034	32,3 ± 5,4^a^	(22,0–42,0)	0,200	<0,001 (U de Mann-Whitney)	–
Talla, cm	145±8^a^	(129–161)	0,200	138±9^a^	(119–155)	0,200	<0,001 (T Student)	0,521
IMC	25,9 ± 3,3^a^	(20,1–36,0)	0,200	17,1 ± 2,4^b^	(13,7–19,9)	0,023	<0,001 (U de Mann-Whitney)	–

	**Mediana**	**Límites**		**Mediana**	**Límites**			

Z-score peso	2,55	(0,63–7,25)	–	−0,56	(−1,75–0,50)	–	–	–
Z-score talla	0,99	(-1,15–3,49)	–	−0,31	(−2,77–2,09)	–	–	–
Z-score IMC	2,80	(0,80–6,31)	–	−0,62	(−1,65–0,34)	–	–	–

^a^Media ± desviación estándar; ^b^mediana (Q1–Q3); ^c^prueba de Kolmogorov-Smirnov con modificación de Lillefors; ^d^comparación de la distribución de la variable sexo en los dos grupos mediante el test de χ^2^ de Pearson (p=0,367).

El peso, la talla y el IMC fueron más elevados, de forma estadísticamente significativa, en el grupo de niños con sobrepeso/obesidad (p<0,001).

Los resultados analíticos de los parámetros estudiados y los obtenidos tras la aplicación de los distintos test estadísticos se muestran en las Tablas 2 y 3.

**Tabla 2:** Tipo de distribución y estudio comparativo parámetros lipídicos y concentración de insulina en niños con sobrepeso/obesidad vs. niños normopeso.

Parámetros lipídicos	Sobrepeso/Obesos		Normopeso		Significación estadística (test)^d^	Valor p test de Levene
	Valor	Pruebas de normalidad^c^	Valor	Pruebas de normalidad^c^		
Colesterol total, mg/dL	167,1 ± 32,6 (158,8–175,5)^a^	0,200	174,0 ± 24,6 (166,6–181,4)^a^	0,200	0,215 (Welch)	0,017
Colesterol LDL, mg/dL	98,7 ± 27,3 (91,8–105,7)^a^	0,194	100,2 ± 18,5 (94,6–105,7)^a^	0,073	0,747 (Welch)	0,003
Triglicéridos, mg/dL	73,0 (61,5–98,5)^b^	<0,001	56,0 (45,5–69,9)^b^	0,004	**<0,001 (U de Mann-Whitney)**	–
Colesterol HDL, mg/dL	51,1 ± 9,6 (48,7–53,6)^a^	0,200	61,6 ± 13,8 (57,4–65,7)^a^	0,200	**<0,001 (Welch)**	0,012
Colesterol no-HDL, mg/dL	116 ± 31 (107–124)^a^	0,171	112 ± 19 (107–118)^a^	0,175	0,473 (Welch)	0,001
Apo A1, mg/dL	148,1 ± 18,7 (143,3–152,9)^a^	0,182	172,6 ± 30,8 (163,3–181,8)^a^	0,200	**<0,001 (Welch)**	0,005
Apo B, mg/dL	81,5 ± 23,3 (75,5–87,4)^a^	0,200	76,6 ± 15,3 (72,0–81,2)^a^	0,180	0,195 (Welch)	0,002
Lipoproteína (a), mg/dL	14,5 (5,37–39,60)^b^	<0,001	10,7 (6,42, 54,80)^b^	<0,001	0,924 (U de Mann-Whitney)	–
Insulina, μUI/mL	11,83 (8,95–17,15)^b^	<0,001	6,60 ± 2,71 (5,78–7,43)^a^	0,200	<0,001 (U de Mann-Whitney)	

^a^Media ± desviación estándar (IC, 95 %); ^b^mediana (Q1–Q3); ^c^Kolmogorov-Smirnov con modificación de Lillefors; ^d^los valores en negrita hacen referencia a los parámetros donde las pruebas de significación estadística mostraron una diferencia estadísticamente significativa entre los dos grupos para un nivel de confianza del 95 %. Apo A1, apolipoproteína A1; Apo B, apolipoproteína B.

**Tabla 3:** Tipo de distribución y estudio comparativo índices lipídicos y de resistencia a insulina en niños con sobrepeso/obesidad vs. niños normopeso.

Ratios e índices	Niños sobrepeso/obesidad		Niños normopeso		Significación estadística (test)^d^	Valor p test de Levene
	Valor	Pruebas de normalidad^c^	Valor	Pruebas de normalidad^c^		
Ratio Apo B/Apo A1	0,56 ± 0,19 (0,51–0,61)^a^	0,099	0,46 ± 0,11 (0,43–0,49)^a^	0,200	**0,001 (Welch)**	0,008
Ratio TG/colesterol HDL	1,44 (1,14–2,05)^b^	<0,001	0,88 (1,41–0,66)^b^	0,001	**<0,001 (U de Mann-Whitney)**	–
Índice colesterol LDL/Apo B	1,23 ± 0,18 (1,18–1,27)^a^	0,188	1,32 ± 0,14 (1,28–1,36)^a^	0,068	**0,004 (T Student)**	**0,215**
Índice TyG	8,15 ± 0,45 (8,03–8,26)^a^	0,200	7,73 ± 0,11 (7,63–7,84)^a^	0,200	**<0,001 (T Student)**	**0,053**
Índice HOMA	2,64 (1,86–3,81)^b^	<0,001	1,40 ± 0,61 (1,20–1,61)^a^	0,200	**<0,001 (U de Mann-Whithey)**	–
Índice QUICKI	0,33 ± 0,03 (0,32–0,34)^a^	0,200	0,37 (0,35–0,39)^b^	0,035	**<0,001 (U de Mann-Whithey)**	–
Partículas remanentes, mg/dL	14 (12–19,5)^b^	<0,001	11 (9–14)^b^	<0,001	**<0,001 (U de Mann-Whitney)**	–

^a^Media ± desviación estándar (IC, 95 %); ^b^mediana (Q1–Q3); ^c^Kolmogorov-Smirnov con modificación de Lillefors; ^d^los valores en negrita hacen referencia a los parámetros donde las pruebas de significación estadística mostraron una diferencia estadísticamente significativa entre los dos grupos para un nivel de confianza del 95 %. TG, triglicéridos; Apo A1, apolipoproteína A1; Apo B, apolipoproteína B; TyG, triglicéridos-glucosa.

Se encontraron diferencias, estadísticamente significativas, entre el grupo de niños con sobrepeso/obesidad y el grupo de niños normopeso en la concentración de TG  y cHDL, siendo más elevada la concentración de TG  y  más baja la de cHDL en el grupo de niños con sobrepeso/obesidad (p<0,001, en ambos casos).

La concentración de partículas remanentes también fue más elevada, de forma estadísticamente significativa (p<0,001), en el grupo de niños con sobrepeso/obesidad.

En cuanto a las apolipoproteínas, encontramos concentraciones más elevadas de Apo A1 en el grupo de niños normopeso, frente al grupo de niños con sobrepeso/obesidad (p<0,001) y la concentración de Apo B fue mayor en el grupo de niños con sobrepeso/obesidad, pese a que no se alcanzó significación estadística (p=0,195). También fue más elevada, en el grupo de niños normopeso, la ratio cLDL/Apo B (p=0,004) y más bajo el Índice TyG y la ratio Apo B/Apo A1 (p<0,001).

La concentración de insulina estaba también más elevada, de forma estadísticamente significativa, en el grupo de niños con sobrepeso/obesidad respecto a la que presentaban los niños normopeso. Los índices HOMA y TyG fueron más elevados y el de QUICKI más bajo en el grupo de niños con sobrepeso/obesidad, (p<0,001, en ambos casos).

**Figura 2:**
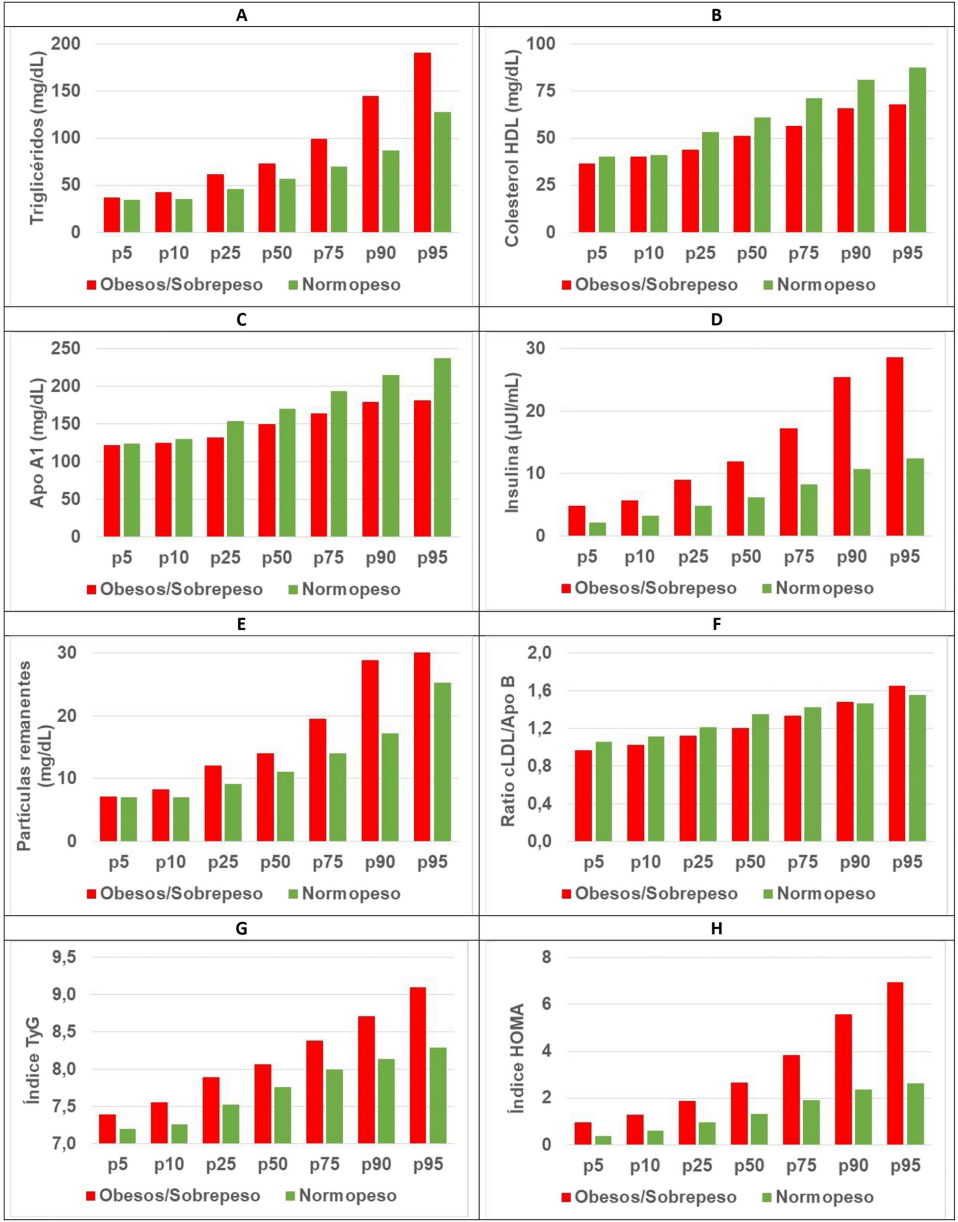
Percentiles de los parámetros e índices lipídicos y hormonales en niños normopeso y con sobrepeso/obesidad. (A) Triglicéridos, (B) colesterol HDL, (C) Apo A1, (D) insulina, (E) partículas remanentes, (F) ratio cLDL/Apo B, (G) índice TyG, (H) índice HOMA. LDL, colesterol LDL; Apo A1, apolipoproteína A1; Apo B, apolipoproteína B; TyG, índice triglicéridos-glucosa.

Los resultados de los percentiles p5, p10, p25, p50, p75, p90 y p95 calculados en aquellos parámetros, cocientes o índices en los que se observaron diferencias significativas entre ambos grupos se encuentran representados en la Figura 2.

Cuando subdividimos los grupos de niños con sobrepeso/obesidad y normopeso en dos subgrupos, según el sexo, en el grupo de niños con sobrepeso/obesidad no se encontraron diferencias estadísticamente significativas en ninguno de los parámetros y cocientes estudiados y en los niños normopeso solamente encontramos diferencias en el índice TyG (niños: 7,66 ± 0.33; niñas: 7,88 ± 0,27; p=0,041 (T Student)). Entre los niños/as con sobrepeso y los niños/as con obesidad no se encontraron diferencias significativas en ninguno de los parámetros y cocientes estudiados.

Se encontró correlación negativa entre el IMC y Apo A1 (r=−0,460; p<0,001) y entre IMC y cHDL (r=−0,433; p<0,001) y positiva entre IMC y  TG (r=0,396; p<0,001) e insulina (r=0,683; p<0,001). Respecto a la edad, se encontró correlación positiva, en el grupo de niños normopeso, con la insulina (r=0,332; p=0,027) y con la lp(a) (r=0,349; p=0,019). En cuanto al sexo no se encontró correlación significativa entre ninguno de los parámetros estudiados.

También se encontró correlación negativa entre el IMC y las ratios/índices cLDL/ApoB (r=−0,238; p=0,014) y QUICKI (r=−0,677; p<0,001) y positiva entre el IMC y ApoB/ApoA1 (r=0,277; p=0,004), TG/cHDL (r=0,461; p<0,001), TyG (r=0,467; p<0,001), HOMA (r=0,677; p<0,001) y partículas remanentes (r=0,397; p<0,001). Respecto a la edad, únicamente se encontró correlación positiva, en el grupo de niños normopeso, con HOMA (r=0,325; p=0,050) y con TyG (r=0,450; p=0,005) y negativa con QUICKI (r=−0,370; p=0,024) y en cuanto al sexo se encontró correlación positiva en el sexo femenino con el índice TyG (r=0,274; p=0,044), solo también en el grupo de niños normopeso.

## Discusión

Según los resultados de nuestro estudio los niños con sobrepeso/obesidad presentan un perfil lipídico claramente más aterogénico que los niños normopeso, con concentraciones más elevadas de TG  y  más bajas de cHDL y de Apo A1_,_ de forma estadísticamente significativa, con respecto al grupo de niños normopeso. Estos resultados coinciden también con los reportados por algunos otros autores [15], 16], en los cuales los niños con sobrepeso/obesidad presentaban concentraciones superiores de TG e inferiores de cHDL y de Apo A1 a las de los niños normopeso. En el estudio de Nielsen y cols. [17], además, en los niños con sobrepeso/obesidad la concentración de cLDL, no-cHDL y de CT (en este caso únicamente en los niños) fue superior a la de los niños normopeso, a diferencia de nuestro estudio, en el cual no encontramos diferencias significativas en estos parámetros. En cuanto a la correlación de los parámetros estudiados con el IMC, al igual que en el estudio de Falaschetti y cols [18]. encontramos correlación negativa de Apo A1 y cHDL con el IMC y la concentración de Apo B también era más alta en el grupo de niños con sobrepeso, aunque en nuestro caso no llegamos a encontrar significación estadística.

Por otra parte, además de los parámetros lipídicos básicos, las lipoproteínas ricas en triglicéridos y las resultantes de su metabolización juegan un papel importante en el riesgo residual de la ECV ya que son altamente aterogénicas por su capacidad de penetrar y ser retenidas en la pared arterial, su alto contenido en colesterol y su capacidad de generar células espumosas. Estas partículas, denominadas partículas remanentes, que incluyen los remanentes de quilomicrones, los de las lipoproteínas de muy baja densidad (VLDL) y los de las lipoproteínas de densidad intermedia (IDL) están relacionadas con el desarrollo de la enfermedad cardiovascular aterosclerótica [19], 20].

En el grupo de niños con sobrepeso/obesidad hemos encontrado mayor concentración de partículas remanentes que en el grupo de los niños normopeso, al igual que en el estudio de Slyper y cols. [21], lo que pone de manifiesto que ya existe en este grupo de niños/as un riesgo incrementado para el desarrollo de la enfermedad cardiovascular aterosclerótica.

En referencia a la concentración de Apo A1 y Apo B, en un metaanálisis del año 2020 en el que se incluyeron cuatro artículos y un total de 7,974 niños y adolescentes, se observó un aumento en la media de Apo A1 en el grupo de niños normopeso, respecto al grupo de niños con sobrepeso u obesidad de 8,13 mg/dL frente a 24,4 mg/dL encontrado por nosotros y se encontraron diferencias similares entre ambos grupos en la concentración de Apo B [22].

Por otra parte, como ya se ha expuesto anteriormente los cocientes, tanto TG/cHDL como Apo B/Apo A1, fueron más elevados en los niños con sobrepeso/obesidad que en los niños normopeso, mientras que el cociente cLDL/Apo B fue más bajo en los niños con sobrepeso/obesidad.

El cociente Apo B/Apo A1 se considera un predictor importante de enfermedad coronaria en pacientes adultos obesos [23] y en diversos estudios realizados en población pediátrica se ha encontrado correlación positiva entre este cociente y la obesidad, además de asociarse con un mayor riesgo cardiovascular [24], 25], resultados acordes con nuestro estudio en el cual los niños con sobrepeso/obesidad tenían valores superiores del ratio Apo B/Apo A1 y por lo tanto, su determinación puede ayudarnos a identificar de forma temprana los niños obesos que presentan más riesgo para el desarrollo de ECV y aterosclerosis.

El cociente cLDL/Apo B determina, de forma indirecta, el número de partículas de lipoproteínas de baja densidad (LDL) pequeñas y densas, que son más aterogénicas que el resto de subclases de partículas LDL [26], 27]. Se ha establecido que un cociente de 1.2 se corresponde con un tamaño de 25.5 nm para las partículas LDL y es además el punto de corte que permite establecer un patrón de LDL pequeñas y densas, siempre que existan valores inferiores o iguales a este punto de corte [28]. Sin embargo, aunque su utilidad para predecir eventos cardiovasculares con enfermedad aterosclerótica [29] y su relación con el SM [30] han sido claramente demostradas en pacientes adultos, en población pediátrica no se tienen todavía suficientes datos sobre su utilidad. Según los resultados de nuestro estudio podemos afirmar que los niños con sobrepeso/obesidad presentan mayor cantidad de partículas LDL pequeñas y densas y por tanto un patrón más aterogénico. Aproximadamente el 50 % de los niños de este grupo tiene un patrón de LDL pequeñas y densas frente a aproximadamente el 25 % en el grupo de los niños normopeso. En el estudio de Xiao y cols [31] realizado sobre población adulta, los pacientes que fallecieron por causas cardiovasculares presentaban un cociente cLDL/ApoB inferior a 1.2  y menor al que presentaban los pacientes que no fallecieron por eventos cardiovasculares, por lo que sería interesante realizar estudios adicionales que nos permitan evaluar su poder predictivo en población pediátrica.

En cuanto al cociente TG/cHDL, diversos estudios han demostrado también su utilidad para predecir en niños con sobrepeso/obesidad la resistencia a la insulina (RI) y el SM [32], [33], [34]. En nuestro estudio, este cociente fue mayor en los niños con sobrepeso/obesidad, lo que supondría un riesgo aumentado de presentar RI en este grupo. Este cociente, junto con los índices HOMA y QUICKI, puede ser útil para identificar de forma precoz aquellos niños con sobrepeso/obesidad con mayor RI y, por lo tanto, mayor riesgo para el desarrollo de DM2.

Asimismo, tanto la concentración de glucosa como la de insulina, el índice HOMA y el índice TyG fueron superiores en el grupo de niños con sobrepeso/obesidad, mientras que el índice de QUICKI fue superior en los niños normopeso.

El índice HOMA representa una estimación de la RI a partir de la medición de glucosa e insulina en ayunas [35], en el cual los valores más altos se relacionan con mayor RI [36], por lo que en nuestro estudio el grupo de niños con sobrepeso/obesidad presenta mayor RI que el grupo de los niños normopeso. Teniendo en cuenta los puntos de corte establecidos para RI asociado al SM, en un metaanálisis del año 2019 [37] se concluyó que estos puntos de corte oscilaban entre 2,30 y 3,54 para niños y adolescentes. Según los percentiles establecidos para HOMA en nuestro estudio, dentro el grupo de niños normopeso, solo el 10 % de estos niños presentaría RI, mientras que en el grupo de niños con sobrepeso/obesidad el 50 % presentarían RI. En el estudio de Mastroeni y cols. [38], similar al nuestro, los niños con sobrepeso/obesidad también presentaban mayor concentración de insulina y mayor índice HOMA.

El índice QUICKI se puede emplear también para estimar la RI, aunque su relación con el método “gold estándar” (clamp hiperinsulinémico-euglucémico) es algo peor que en el caso del índice HOMA [39]. En nuestro estudio, los niños con sobrepeso/obesidad presentaban valores inferiores a los niños normopeso, resultados acordes a los encontrados en el índice HOMA, donde los niños con sobrepeso/obesidad presentaban mayor RI. Asimismo, en el estudio de Sapunar y cols [40]. los niños con sobrepeso/obesidad presentaron también valores superiores de HOMA e inferiores de QUICKI en comparación con los niños normopeso.

El grupo de niños con sobrepeso/obesidad presentó también valores más elevados del índice TyG. Este índice al igual que los índices HOMA y QUICKI se relaciona con el desarrollo de RI [41], sirviendo también como indicador para evaluar el riesgo de llegar a presentar SM [42]. En la actualidad, se han llegado a determinar distintos puntos de corte de TyG en diferentes poblaciones para predicción de la insulinoresistencia con respecto al índice HOMA, así, en el estudio de Dikaiakau y cols. el punto de corte de dicho índice más adecuado para predecir RI es 7,91, por lo que teniendo en cuenta este punto de corte, en nuestro estudio cerca del 75 % de los niños con sobrepeso/obesidad podrían presentar RI, en contraste con el grupo de niños normopeso en el cual sobre el 25 % podría presentar RI. También hemos podido comprobar en nuestro estudio que los niños con IMC más elevado presentan un perfil lipídico más aterogénico y mayor RI. En cuanto a la edad y al sexo se observaron en el grupo de niños normopeso índices HOMA y TyG más altos y el índice QUICKI más bajo al aumentar la edad, siendo esta correlación más débil que con el IMC, así como una correlación ligeramente significativa del índice TyG con el sexo, donde las niñas presentaban valores ligeramente superiores que los niños. Estos resultados coinciden con los encontrados en otros estudios en los cuales en los niños normopeso a medida que aumenta su edad también aumenta el índice HOMA y disminuye el índice QUICKI [35], 43].

Como limitaciones de nuestro estudio podríamos considerar el escaso tamaño muestral y como fortalezas el haber partido de dos grupos de niños muy bien caracterizados y en edad prepuberal, en la cual no es muy frecuente este tipo de estudios. Un aspecto interesante, sería poder ampliar nuestro tamaño muestral, para poder llegar a establecer una diferenciación entre niños/as con sobrepeso y niños/as con obesidad, la influencia de la edad y sexo, así como estudiar otra serie de parámetros lipídicos más sensibles que nos permitan llegar a conocer con mayor precisión el riesgo metabólico y cardiovascular de estos niños.

Como conclusión podemos decir que nuestro grupo de niños con sobrepeso/obesidad de edades comprendidas entre 8  y 12 años presentan un perfil lipídico más \aterogénico, mayor concentración de partículas remanentes y de partículas LDL pequeñas y densas, todas ellas muy aterogénicas, así como una mayor insulinoresistencia en comparación con los niños normopeso, lo que podría implicar un mayor riesgo de desarrollar DM2 y ECV en los niños con sobrepeso/obesidad.
